# Spinal Cord Stimulation for Refractory Chest Pain Without Objective Ischemia in a Patient With Prior Coronary Artery Bypass Grafting: A Case Report

**DOI:** 10.7759/cureus.104195

**Published:** 2026-02-24

**Authors:** Jad Kabbara, Cham Al Salak, Humza Arif, Abdallah Kabbara

**Affiliations:** 1 Anesthesiology, Lake Erie College of Osteopathic Medicine, Erie, USA; 2 Anesthesiology, Damascus University, Damascus, SYR; 3 Internal Medicine, Lake Erie College of Osteopathic Medicine, Erie, USA; 4 Anesthesiology, University Hospitals Cleveland Medical Center, Cleveland, USA

**Keywords:** neuromodulation, nonischemic chest pain, opioid-sparing analgesia, refractory angina, spinal cord stimulation (scs)

## Abstract

Refractory nonischemic chest pain in patients with prior coronary artery bypass grafting (CABG) and optimal medical therapy presents a significant management dilemma when conventional cardiac evaluations are nondiagnostic. We report a case of a 60-year-old male patient with prior CABG and multiple percutaneous coronary interventions who experienced recurrent admissions for severe chest pain despite angiographically patent grafts and nondiagnostic ischemic evaluations, requiring escalating opioid therapy for symptom control. Under fluoroscopic guidance, percutaneous epidural leads were placed at T6-T7 to target the T2-T4 cardiac dermatomes. During a four-day trial of spinal cord stimulation (SCS), he experienced complete resolution of chest pain, discontinued opioids, and returned to full functional capacity. Permanent implantation was performed without complications. At six-month follow-up, he remained pain-free (0/10), had no further hospital readmissions, and reported marked improvement in quality of life. We propose that SCS may provide analgesia through neuromodulation of dorsal column pathways and partial sympathetic tone modulation of cardiac accelerator fibers, optimizing myocardial oxygen supply-demand balance. This case highlights SCS as a promising analgesic strategy for refractory nonischemic chest pain in anesthesia practice.

## Introduction

Chest pain that persists despite coronary revascularization and optimized medical therapy remains clinically challenging, particularly when repeated evaluations do not demonstrate active myocardial ischemia. The term refractory angina pectoris is generally reserved for chronic angina symptoms with objective evidence of ischemia that persist despite optimized medical therapy and when further revascularization is not feasible [1]. In contrast, refractory chest pain without objective ischemia describes persistent symptoms in which serial testing fails to confirm ongoing ischemia, even in patients with extensive prior coronary disease and revascularization. Patients with refractory angina often experience limitations in daily activities and a decreased quality of life due to constant symptoms. The American College of Cardiology specifies the parameters for refractory angina pectoris into three specific categories: (i) anginal chest pain despite intensive medical therapy; (ii) objective evidence that symptoms are of ischemic origin; and (iii) further options of coronary revascularization have been deemed unfeasible [1]. Recent literature has shown that refractory angina can occur in both obstructive epicardial coronary artery disease and nonobstructive mechanisms, such as vasospasms, even if revascularization therapy appears successful [2]. Importantly, refractory angina refers to persistent symptoms attributable to myocardial ischemia with objective evidence of ischemia. In contrast, chronic noncardiac or nonischemic chest pain describes persistent chest pain in which repeated evaluations do not demonstrate active ischemia, even in patients with prior coronary revascularization.

The principal objective in the management of refractory angina pectoris is the alleviation of symptoms to enhance patients' quality of life. Although symptom management remains the cornerstone of treatment, alternative approaches are often sought to avoid long-term opioid use in patients with refractory chest pain and angina, given the limited efficacy of opioids in these cases, which can lead to opioid dependence without resolving the underlying issue. In this case of a 60-year-old Caucasian man whose cardiac status had been fully optimized, continued opioid use was deemed suboptimal. As a result, alternative strategies for managing his chronic chest pain were explored. This led to the implementation of spinal cord stimulation (SCS) targeting the thoracic spinal segments T2-T4 in an effort to provide effective analgesia. This intervention resulted in sustained pain relief and a significant improvement in quality of life, successfully eliminating the need for opioid therapy.

## Case presentation

A 60-year-old man presented with a history of coronary artery disease status post coronary artery bypass grafting (CABG), multiple prior percutaneous coronary interventions with stent placement, and cardiovascular comorbidities, including hypertension (HTN). His chronic medications included a beta-blocker, nitrates, and a calcium channel blocker (CCB). He experienced multiple emergency visits/admissions for severe substernal chest pain rated up to 9/10, frequently requiring intravenous opioid therapy. Noncardiac contributors were considered, including gastroesophageal reflux disease (GERD), and he had trialed prior therapies such as gabapentin without sustained relief.

The patient was referred for evaluation following multiple admissions to the coronary care unit for recurrent chest pain. Comprehensive cardiac workups, including imaging and laboratory investigations, consistently excluded cardiac etiology. Despite a history of CABG and multiple stent placements, the patient had been optimized from a cardiology standpoint but continued to require escalating doses of opioids for pain control. The patient expressed significant distress regarding recurrent hospitalizations and functional impairment and requested an opioid-sparing long-term strategy.

**Figure 1:**
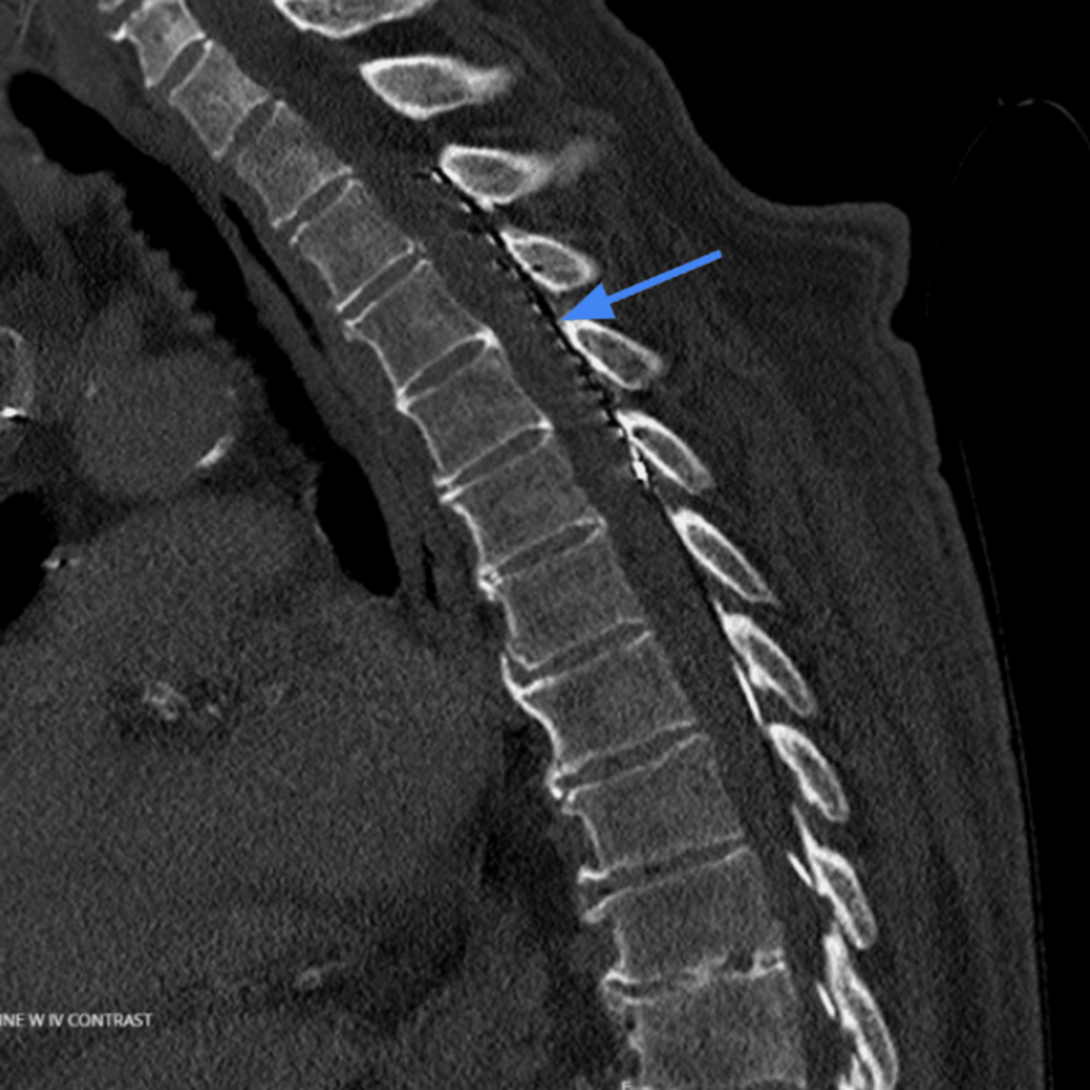
Intraprocedural fluoroscopic image demonstrating percutaneous spinal cord stimulation leads (arrow) positioned within the thoracic epidural space. The lead tips were advanced cephalad to approximate the T2-T4 vertebral levels, corresponding to the patient’s anterior chest wall pain distribution. Vertebral body landmarks were visible to assist in level identification. Final positioning was confirmed intraprocedurally using both anteroposterior and lateral fluoroscopic views.

**Figure 2:**
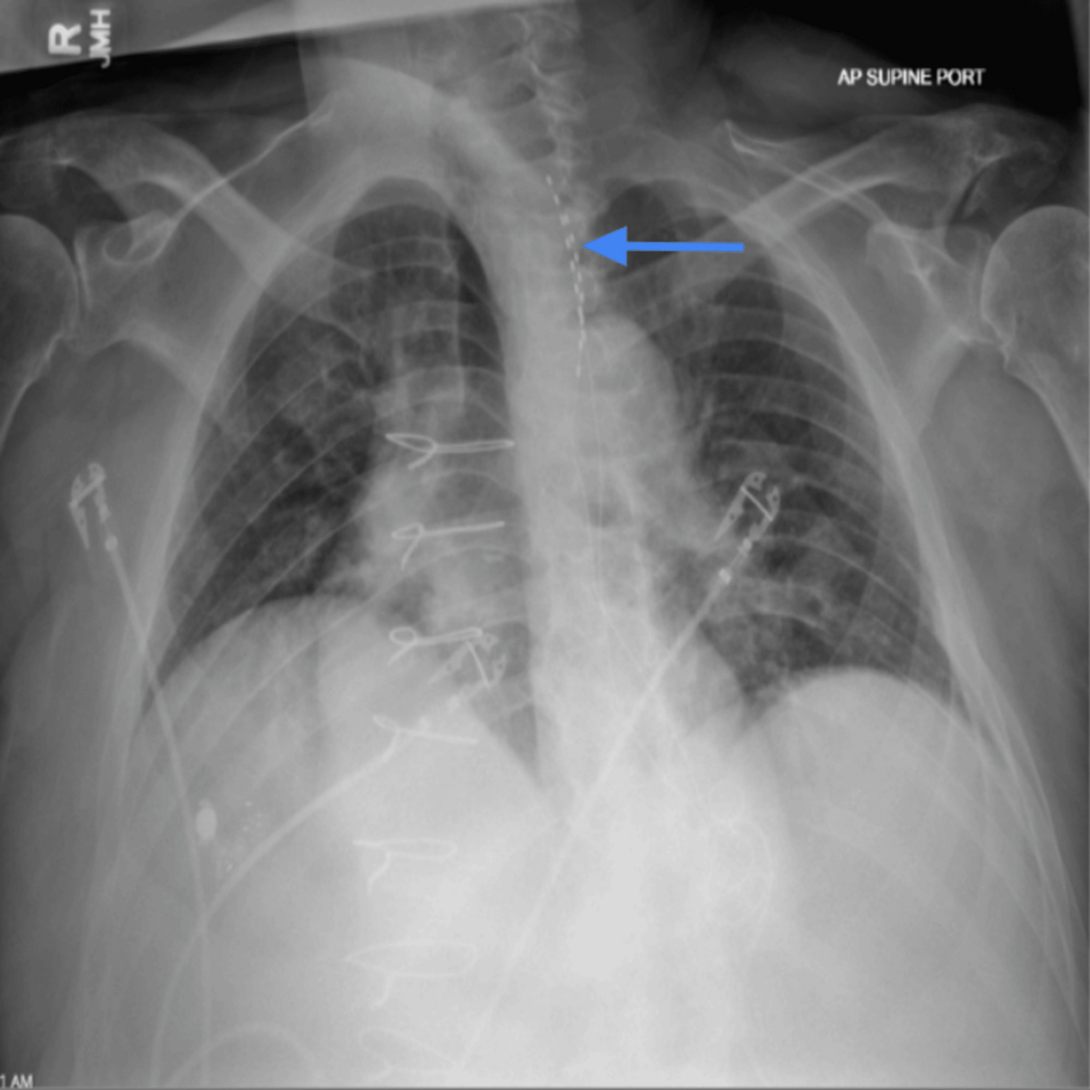
Anteroposterior chest radiograph obtained during the spinal cord stimulation trial demonstrating the projected location of the percutaneous leads (arrow) over the upper thoracic spine. Mild rotational artifact may have limited precise assessment of strict midline orientation on this view alone. Posterior epidural location and final lead positioning were confirmed intraprocedurally using orthogonal fluoroscopic imaging.

Upon interview, the patient appeared frustrated by his repeated hospitalizations, expressing concern over missed work obligations and a strong willingness to pursue any intervention that could alleviate his symptoms. A trial of SCS was proposed. The thoracic spine was accessed at the T6-T7 level using a normal saline loss-of-resistance technique under fluoroscopic guidance. Two percutaneous eight-contact Medtronic Octrode leads (Model 977A190; Medtronic, Minneapolis, USA) were placed in the thoracic epidural space under fluoroscopic guidance (Figures 1-2). The leads were advanced cephalad and positioned to approximate the T2-T4 vertebral levels corresponding to the patient’s anterior chest wall pain distribution. Final positioning was confirmed intraprocedurally using both anteroposterior and lateral fluoroscopic views. Trial stimulation was delivered using SCS parameters consisting of a pulse width of 420 microseconds, frequency of 50 Hz, and amplitude of 2 mA. These settings achieved comfortable paresthesia coverage overlapping the anterior chest wall corresponding to the patient’s pain distribution. The patient reported complete pain relief during the four-day trial. Following permanent implantation, stimulation parameters remained unchanged from the successful trial configuration.

The patient was discharged with a four-day trial of SCS. During the trial period, he reported complete resolution of his chest pain and cessation of opioid use. He expressed a desire to retain the leads permanently; however, due to the risk of infection and his need for anticoagulation therapy, the leads were removed at the conclusion of the trial. A permanent spinal cord stimulator (Medtronic, Minneapolis, USA) was subsequently implanted using the same technique. Permanent implantation was performed using a percutaneous technique consistent with the successful trial configuration. The epidural leads were positioned at the same thoracic levels to reproduce effective dermatomal coverage. The leads were secured and tunneled subcutaneously to an implantable pulse generator (IPG) (Medtronic, Minneapolis, USA) placed in a right flank subcutaneous pocket. The procedure was completed without intraoperative complications. Programming parameters following permanent implantation remained consistent with the successful trial settings without significant modification.

At the six-month follow-up post-implantation, the patient remained symptom-free with no further hospitalizations for chest pain. He remained off opioid therapy and reported sustained improvement in his quality of life, consistently rating his pain as 0 out of 10. No dedicated post-implant cardiac stress testing or repeat coronary angiography was performed, as the patient remained clinically stable without recurrent ischemic symptoms. Functionally, he returned to work and reported a threefold expansion in the size of his business.

## Discussion

This case illustrates the effective use of SCS for the treatment of refractory nonischemic chest pain in patients with prior patent grafts or CABG surgery. Our patient experienced complete resolution of his refractory chest pain and was able to sustain functional recovery. Recent evidence supports SCS as a form of treatment in patients with refractory angina pectoris or those with chest pain unresponsive to conventional therapies. A prospective single-center cohort study demonstrated the long-term efficacy and improved quality of life over an average follow-up time of 69 months in refractory angina pectoris patients who underwent SCS therapy [3]. Specifically, the study noted a reduced frequency of anginal episodes, nitroglycerin use, and an overall improved exercise capacity and quality of life in SCS-treated patients. These findings align with our patient's outcomes who experienced symptomatic relief, opioid cessation, and an increase in functional quality of life. Although SCS has most commonly been studied in refractory ischemic angina populations, this case illustrates its potential opioid-sparing role in a post-revascularization patient with refractory chest pain without objective ischemia after extensive evaluation.

The mechanism of SCS benefits in refractory angina pectoris is through reduced sympathetic modulation, which leads to decreased myocardial oxygen demand and redistribution of myocardial perfusion. Dorsal column stimulation for intractable angina was described in early reports in the late 1980s, including by Murphy and Giles (1987), and has since been evaluated across multiple observational studies and trials in refractory angina cohorts [4]. As summarized by a systematic review conducted by Gazzeri et al., the effect of SCS on refractory angina pectoris occurs through activation of large-diameter Aβ fibers in the dorsal column, which dampen nociceptive C-fiber signaling in the dorsal horn, reducing the perception of chest pain [5]. SCS has also been effective in decreasing serotonergic and cholinergic-mediated pathways, which further diminishes nociceptive transmission at the spinal level, decreasing the sensation of pain [6]. Additionally, a study conducted by Wang et al. found that SCS suppressed spinal dorsal horn microglial activation via the p38 MAPK pathway, resulting in reduced IL‑1β and TNF‑α release, thereby decreasing inflammation and pain [7]. SCS has been reported to modulate sympathetic activity, which may reduce cardiac sympathetic drive and improve myocardial oxygen supply-demand balance, thereby alleviating ischemic chest discomfort in selected patients [5].

SCS has been reported with very minimal complication rates, with infection rates under 11% and lead fractures occurring in less than 8% of cases [8]. There has been no increase in mortality or masking of ischemic warning signals observed in patients during long-term follow-up [9-12]. In our case, neither trial nor permanent implantation resulted in any procedural complications.

Our patient’s clinical improvement reinforces SCS as a potential third-line intervention for refractory angina pectoris in nonischemic cardiac patients who are unresponsive to conventional measures. This case supports the concept of individualized patient selection, taking into account risk factors, anticoagulation needs, and device-related considerations for individuals wishing to undergo SCS [13,14]. As a single-patient case report, these findings should be interpreted cautiously and are not intended to imply universal efficacy. What this case adds is a detailed, clinically pragmatic account of dermatomal targeting for anterior chest pain coverage, opioid discontinuation following SCS in a post-CABG patient without objective ischemia, and the decision-making process surrounding temporary trial removal and subsequent permanent implantation in the context of required antithrombotic therapy.

## Conclusions

SCS is a recognized treatment option for refractory nonischemic chest pain in patients with prior CABG and optimal medical therapy who continue to experience severe, disabling symptoms despite patent grafts and stents. SCS can provide significant symptomatic relief, reduce angina frequency, improve quality of life, and decrease opioid requirements in this population, with a favorable safety profile and no increase in mortality risk. In patients who are not candidates for further revascularization, SCS is associated with sustained reductions in anginal episodes and hospital admissions, as well as improvements in functional status and quality of life, as shown in prospective registries and systematic reviews. 

The mechanism is thought to involve modulation of cardiac sympathetic fibers and improvement of myocardial oxygen supply-demand balance. SCS is considered a first-line adjunctive therapy for refractory angina in patients who have exhausted conventional options, and its use is supported by strong evidence for efficacy and safety in this context. Careful patient selection and multidisciplinary management are essential to optimize outcomes.
